# Edinburgh University Solution of Lime (EUSOL) Versus Platelet-Derived Growth Factor Dressing for Wound Contraction in Grade II Wagner Diabetic Foot Ulcers: A Randomized Controlled Trial

**DOI:** 10.7759/cureus.114872

**Published:** 2026-08-20

**Authors:** Fizza Batool, Fasiha Munawwar, Hassan Shaukat, Atif A Janjua, Umar Niazi, Shabbar H Changazi

**Affiliations:** 1 General Surgery, Hameed Latif Hospital, Lahore, PAK; 2 General Surgery, Services Hospital, Lahore, PAK; 3 General Surgery, Lahore general hospital, Lahore, PAK; 4 General Surgery, Lahore General Hospital, Lahore, PAK; 5 General Surgery, Provincial Headquarter Teaching Hospital, Gilgit, PAK

**Keywords:** antiseptic dressing, diabetic foot ulcer, eusol, platelet-derived growth factor, randomized controlled trial, wagner grade ii, wound contraction, wound healing

## Abstract

Background

Diabetic foot ulcers remain a leading cause of lower-limb amputation worldwide, and wound management is especially difficult in resource-limited settings where older antiseptic agents such as Edinburgh University Solution of Lime (EUSOL) are still in routine use. This trial compared EUSOL dressing with platelet-derived growth factor (PDGF) dressing for wound contraction in Grade II Wagner diabetic foot ulcers.

Methodology

In this randomized controlled trial, 120 patients aged 30 to 70 years with Grade II Wagner diabetic foot ulcers were assigned in a 1:1 ratio to daily EUSOL dressing or PDGF dressing applied every fourth day for four weeks after debridement and infection control. Wound healing was defined as a reduction in ulcer area of at least 50% from baseline at four weeks. A multivariate logistic regression model examined treatment group, age, sex, body mass index (BMI), diabetes duration, and antidiabetic therapy as predictors of healing.

Results

Patients averaged 49.8 ± 10.2 years of age and a BMI of 27.1 ± 3.45 kg/m²; 58.3% were male. Seventy-eight patients (65%) used oral hypoglycemic agents alone, while 42 (35%) required insulin. Seventy-one patients (59.2%) had diabetes for more than 10 years. Wound contraction occurred in 20 of 60 (33.3%) patients on EUSOL versus 44 of 60 (73.3%) on PDGF (*P* < 0.001). PDGF remained a strong independent predictor of healing on multivariate analysis (adjusted odds ratio (OR) 6.14, 95% confidence interval (CI) 2.69-14.02, *P* < 0.001), as did insulin therapy (adjusted OR 2.48, 95% CI 1.10-5.59, *P* = 0.028).

Conclusions

PDGF dressing produced substantially better wound contraction than EUSOL in Grade II diabetic foot ulcers, an effect that persisted after adjustment for the usual confounders. Insulin use was independently linked to better healing, pointing to glycemic management as a modifiable lever alongside dressing choice. Confirmation in multicenter trials with longer follow-up, blinded outcome assessment, and cost data would strengthen the case for wider adoption of PDGF-based dressings in similar settings.

## Introduction

Diabetic foot ulcers (DFUs) affect up to 15% of diabetics over their lifetime, and account for approximately half to two-thirds of the non-traumatic lower extremity amputations performed globally [1,2]. In addition to the possibility of amputation, these ulcers result in prolonged hospitalizations, repeated events, and a true quality-of-life burden. This burden rests disproportionately on low- and middle-income countries, often due to delayed presentation and lack of a multidisciplinary approach to foot care [3].

There is no one single defect in a diabetic wound, but rather multiple defects. Impaired chemotaxis and phagocytosis, together with micro- and macrovascular insufficiency and peripheral neuropathy, create a hostile microenvironment that impedes normal wound healing in diabetic patients [4,5]. Chronic hyperglycemia leads to oxidative stress, which affects fibroblast function, collagen deposition, and new vessel growth. Diabetic ulcers also have a different inflammatory profile than acute wounds, being characterized by a greater chronic inflammatory infiltrate, a shifted cytokine profile, and reduced growth factor activity that is essential to the wound healing process [6,7].

Standard treatment involves the use of sharp debridement, infection control, offloading, glycemic optimization, and appropriate dressing [8]. In low-resource areas, Edinburgh University Solution of Lime (EUSOL) is still widely used as an antiseptic due to its ease of preparation and low cost. The downside is that, in vitro, it is toxic to fibroblasts and keratinocytes, thus raising the concern that it might slow granulation and re-epithelialization in wounds that are already struggling [9]. In contrast, newer dressings and biologic agents are designed to actively modify the wound environment by using scaffolds, cells, or growth factors to remodel the environment around a wound [10].

One such agent is recombinant human platelet-derived growth factor (PDGF). In chronic wounds, it mobilizes fibroblasts and smooth muscle cells and stimulates their proliferation, as well as the stimulation of angiogenesis and matrix deposition [11]. PDGF-containing preparations have been shown to shorten the healing time when compared to placebo and standard care in diabetic ulcers of the lower leg [12,13]. To date, no direct comparison with a traditional antiseptic like EUSOL has been made, and few studies have been conducted in South Asian populations. To fill that gap, this trial compared the wound contraction of EUSOL versus PDGF dressings in Grade II Wagner DFUs and evaluated whether the advantages of PDGF remained after adjusting for age, sex, BMI, diabetes duration, and antidiabetic therapy.

## Materials and methods

This was a randomized controlled trial conducted at the Surgical Department, Services Hospital Lahore, from January 2020 to December 2025, with ClinicalTrials.gov registration number: NCT07493343. The study was conducted in accordance with institutional ethical guidelines, and informed written consent was obtained from all participants before participation.

Sample size was estimated using WHO software for two independent proportions, with a preliminary estimate of 12% healing with EUSOL and 40% healing with PDGF (α = 0.05, two-sided chi-square test) [14], which would provide more than 90% power to detect such a difference with 60 patients per arm.

Patients who visited the outpatient and emergency departments at the hospital and fulfilled the inclusion criteria were selected using non-probability consecutive sampling. All the individuals had diabetes mellitus and were diagnosed with grade II (penetrating subcutaneous tissue but leaving the tendon, bone, and joint intact) diabetic foot ulcer with an ulcer area less than 6 × 6 cm at baseline. Patients were excluded if they had an HbA1c level of >8.5%, an ankle-brachial pressure index of <0.8, immunosuppressive therapy, uncontrolled cardiopulmonary disease, or were unwilling to provide consent. Patients were all systemically or topically treated with antimicrobials for their infection and were subsequently taken to surgery for complete debridement before randomization. Randomization was performed using a computer-generated random number sequence with a 1:1 allocation ratio and a fixed block size of four. Allocation concealment was maintained by placing group assignments in sequentially numbered, opaque, sealed envelopes that were opened only after the completion of debridement and immediately before the first dressing application. The same surgical team measured the length and width of the ulcer with a sterile ruler at baseline and at four weeks following surgery, and the area was calculated as length multiplied by width; this procedure has been shown to correlate with digital planimetry for serial wound assessment. A 50% or greater decrease in the calculated ulcer area from baseline at four weeks was considered wound healing. This threshold aligns with evidence that percentage area reduction at four weeks correlates with eventual complete healing in DFUs. In the prospective study that informed this approach, a reduction of around 53% at four weeks was predictive of eventual complete healing of DFUs [15].

Participants were equalized in group size by block randomization. Group A was given "EUSOL" dressing daily, which was freshly prepared after debridement and cleansing. The topical preparations consisted of a solution of PDGF, which was applied to Group B every fourth day, accompanied by saline dressing changes on the intervening days, while Group A got saline only dressing changes all the time. The treatment for both groups included maintenance of diabetic care, infection management, pressure offloading, and lower limb care, following institutional protocols. Follow-up ran for four consecutive weeks, and the ulcer area was re-measured; percent healing, as compared with baseline, was calculated. The two arms were very different with respect to dressing, so the surgical team who performed outcome measurement was not blinded to treatment allocation; this is a limitation discussed in the Discussion section.

Age, sex, body mass index (BMI), ulcer site, diabetes duration (≤10 years or >10 years), antidiabetic therapy (oral hypoglycemic agents alone or insulin therapy), baseline ulcer area, and four-week healing status were obtained on a structured proforma in addition to treatment group. These same variables were considered as potential confounders of the association between dressing type and wound contraction. The obtained data were analyzed using SPSS version 21 (IBM Corp., Armonk, NY). All continuous variables (age, BMI) were presented as mean ± SD, and comparisons between groups were conducted by independent t-test, while comparisons of categorical variables (sex, healing status) were performed by chi-square test, and the significance was set at *P* ≤ 0.05.

A multivariate logistic regression model was fitted with healing (yes or no) as the outcome and the treatment group, age, sex, BMI, diabetes duration, and the use of antidiabetic therapy as simultaneous predictors to assess the role of the treatment group as an independent predictor of healing. Each predictor has odds ratios with 95 percent confidence intervals. The overall model fit was determined by the likelihood ratio test and the Nagelkerke pseudo *R*².

## Results

One hundred twenty patients with Grade II Wagner diabetic foot ulcers were enrolled, 60 in each group. Mean age was 49.8 ± 10.2 years, and mean BMI was 27.1 ± 3.45 kg/m². Seventy (58.3%) patients were male, and 50 (41.7%) were female, with a similar distribution across groups. Age, sex, and BMI did not differ significantly between groups, confirming adequate baseline comparability (Table 1).

**Table 1:** Baseline characteristics of patients with Grade II Wagner diabetic foot ulcers. Continuous variables were compared with the independent t-test; categorical variables were compared with Pearson's chi-square test. All *P*-values were two-tailed. EUSOL, Edinburgh University Solution of Lime; PDGF, platelet-derived growth factor; BMI, body mass index

Variable	EUSOL (*n* = 60)	PDGF (*n* = 60)	Total (*n* = 120)	Test statistic	*P*-value
Age (years), mean ± SD	49.6 ± 10.3	50.0 ± 10.1	49.8 ± 10.2	*t* = 0.21	0.83
BMI (kg/m²), mean ± SD	27.0 ± 3.48	27.2 ± 3.42	27.1 ± 3.45	*t* = 0.32	0.75
Male sex, *n* (%)	34 (56.7)	36 (60.0)	70 (58.3)	*χ*² = 0.14	0.71
Female sex, *n* (%)	26 (43.3)	24 (40.0)	50 (41.7)	*χ*² = 0.14	0.71
Diabetes >10 years, *n* (%)	34 (56.7)	37 (61.7)	71 (59.2)	*χ*² = 0.31	0.58
Oral hypoglycemics, *n* (%)	39 (65.0)	39 (65.0)	78 (65.0)	*χ*² = 0.00	0.99
Insulin, *n* (%)	21 (35.0)	21 (35.0)	42 (35.0)		

Seventy-eight (65.0%) patients managed their diabetes with oral hypoglycemic agents alone, and 42 (35.0%) required insulin. Seventy-one (59.2%) patients had had diabetes for more than 10 years. Both diabetes duration and type of antidiabetic therapy were evenly distributed between the EUSOL and PDGF groups at baseline.

At four weeks, healing was markedly more common with PDGF: 44 (73.3%) of 60 patients reached the 50% contraction threshold, compared with 20 (33.3%) of 60 on EUSOL (χ² = 19.05, *P* < 0.001; Table 2).

**Table 2:** Wound healing outcomes at four weeks by treatment group (χ² = 19.05). EUSOL, Edinburgh University Solution of Lime; PDGF, platelet-derived growth factor

Outcome	EUSOL (*n *= 60)	PDGF (*n *= 60)	*P*-value
Healed (≥50% contraction), *n* (%)	20 (33.3)	44 (73.3)	<0.001
Not healed (<50% contraction), *n* (%)	40 (66.7)	16 (26.7)	

The multivariate model fit the data well (likelihood ratio χ² = 32.15, *P* < 0.001; Nagelkerke *R*² = 0.31). PDGF treatment carried the strongest association with healing (adjusted OR 6.14, 95% CI 2.69-14.02, *P* < 0.001). Insulin therapy was independently associated with higher odds of healing as well (adjusted OR 2.48, 95% CI 1.10-5.59, *P* = 0.028). Age, BMI, sex, and diabetes duration beyond 10 years showed no independent association with healing, with confidence intervals spanning the null value (Table 3).

**Table 3:** Multivariate logistic regression for predictors of wound healing. PDGF, platelet-derived growth factor; BMI, body mass index; SE, standard error; OR, odds ratio; CI, confidence interval

Predictor	β	SE	Wald χ²	*P*-value	Adjusted OR (95% CI)
Constant	-3.22	2.31	1.94	0.16	-
PDGF group	1.82	0.43	17.92	<0.001	6.14 (2.69-14.02)
Age (years)	0.02	0.02	1.78	0.18	1.02 (0.98-1.06)
BMI (kg/m²)	0.03	0.06	0.25	0.62	1.03 (0.91-1.16)
Male sex	-0.58	0.44	1.74	0.19	0.56 (0.24-1.31)
Diabetes >10 years	0.12	0.45	0.07	0.79	1.13 (0.47-2.71)
Insulin therapy	0.91	0.42	4.70	0.028	2.48 (1.10-5.59)

## Discussion

PDGF dressing outperformed EUSOL by a wide margin in this trial: nearly three-quarters of patients healed on PDGF compared with a third on EUSOL over the same four weeks. That gap is larger than the 12% versus 40% difference used to size the study, which means the observed effect was larger than assumed rather than smaller - reassuring for the trial's power, though it is also worth asking whether a single-center design with unblinded, ruler-based outcome assessment inflated the difference somewhat. This correlation of insulin use and healing continued even after the researchers accounted for type of dressing, age, sex, BMI and diabetes duration. Insulin therapy was independently associated with higher odds of healing, though the mechanisms underlying this association remain uncertain in the absence of objective glycemic data. Furthermore, diabetes duration (of >10 years) was not a significant independent predictor of poor healing, suggesting that duration of diabetes alone is not the obstacle to advanced wound care that is sometimes believed.

These results are similar to earlier studies of PDGF in diabetic ulcers. In Steed's multicenter study, healing was faster and time to closure was shorter with PDGF than placebo [12], and subsequent studies have consistently demonstrated the beneficial effect of exogenous PDGF in wounds that would otherwise stall in granulation tissue formation, new vessel growth, and re-epithelialization [11,13,15]. The four-week endpoint was used here based on the work of Sheehan et al., which showed that patients who healed completely by 12 weeks had already shrunk their wound size by about 50% at four weeks, a significant reduction compared with patients who did not heal [14]. The current findings are consistent with that trend and extend the evidence by providing a direct comparison between PDGF dressing and a conventional antiseptic (EUSOL) in a tertiary care setting in South Asia, a comparison that has not previously been reported.

The limitation of EUSOL is physical, not incidental: it disinfects, but it does not provide any of the scaffolding and any growth factors that a wound needs to regenerate. Laboratory and clinical studies have both raised concern that the use of hypochlorite solutions can harm the fibroblasts, keratinocytes, and endothelial cells, potentially impairing proliferation and matrix formation at the time when it is most needed [9,16]. In some cases, use of traditional antiseptics has even been shown to contribute to delayed wound healing or biologically active wound dressing [9]. Despite its limitations, EUSOL remains widely used in resource-limited settings due to its low cost and lack of specialized storage requirements. This pragmatic reality underscores the importance of the present direct comparison with a growth factor-based alternative, as it provides evidence to guide clinical decision-making in such settings.

Several limitations should be kept in mind. The findings of this study may not be generalizable to other practice settings and populations because it was conducted at a single site. The four-week follow-up period is too short to make any comments regarding complete epithelialization, ulcer recurrence, limb salvage rate, or amputations. Cost data were not collected, which is important for settings selecting an antiseptic near-free product, versus a biologic product. Glycated hemoglobin (HbA1c) levels were not recorded, which precludes a definitive assessment of the relationship between glycemic control and wound healing and limits interpretation of the independent association observed with insulin therapy. The discrepancy in dressing schedules and appearance of arms also prohibited blinding of outcome assessment, which could be overcome by a future trial using an independent blinded assessor or photographic planimetry to measure the outcome, a possible source of bias. To determine the extent to which these results should influence routine diabetic foot care in resource-limited settings, future multicenter trials with blinded outcome assessment and formal cost-effectiveness analysis are needed, with longer follow-up periods.

## Conclusions

PDGF dressing produced substantially better wound contraction than EUSOL dressing in Grade II Wagner diabetic foot ulcers, and this advantage persisted after adjustment for age, sex, BMI, diabetes duration, and antidiabetic therapy. Insulin therapy also predicted better healing, independently of dressing type, indicating that glycemic control should be considered an additional factor to be considered in diabetic foot care. Multicenter, blinded, prospective, longer-term, larger trials with cost-effectiveness data would help to define the role of PDGF-based dressings in pathways of diabetic foot care in resource-limited areas.
